# Enhanced production yields of rVSV-SARS-CoV-2 vaccine using Fibra-Cel^®^ macrocarriers

**DOI:** 10.3389/fbioe.2024.1333548

**Published:** 2024-02-21

**Authors:** Noam Cohen, Irit Simon, Ophir Hazan, Arnon Tal, Hanan Tzadok, Lilach Levin, Meni Girshengorn, Lilach Cherry Mimran, Niva Natan, Tzadok Baruhi, Alon Ben David, Osnat Rosen, Shlomo Shmaya, Sarah Borni, Noa Cohen, Edith Lupu, Adi Kedmi, Orian Zilberman, Avital Jayson, Arik Monash, Eyal Dor, Eran Diamant, Michael Goldvaser, Inbar Cohen-Gihon, Ofir Israeli, Shirley Lazar, Ohad Shifman, Adi Beth-Din, Anat Zvi, Ziv Oren, Arik Makovitzki, Elad Lerer, Avishai Mimran, Einat Toister, Ran Zichel, Yaakov Adar, Eyal Epstein

**Affiliations:** ^1^ Department of Biotechnology, Israel Institute for Biological Research, Ness-Ziona, Israel; ^2^ Department of Organic Chemistry, Israel Institute for Biological, Israel Institute for Biological Research, Ness-Ziona, Israel; ^3^ Department of Biochemistry and Molecular Genetics, Israel Institute for Biological Research, Ness-Ziona, Israel

**Keywords:** VSV, virus vaccine, bioprocess, production, fixed-bed bioreactor, genetic stability

## Abstract

The COVID-19 pandemic has led to high global demand for vaccines to safeguard public health. To that end, our institute has developed a recombinant viral vector vaccine utilizing a modified vesicular stomatitis virus (VSV) construct, wherein the G protein of VSV is replaced with the spike protein of SARS-CoV-2 (rVSV-ΔG-spike). Previous studies have demonstrated the production of a VSV-based vaccine in Vero cells adsorbed on Cytodex 1 microcarriers or in suspension. However, the titers were limited by both the carrier surface area and shear forces. Here, we describe the development of a bioprocess for rVSV-ΔG-spike production in serum-free Vero cells using porous Fibra-Cel^®^ macrocarriers in fixed-bed BioBLU^®^320 5p bioreactors, leading to high-end titers. We identified core factors that significantly improved virus production, such as the kinetics of virus production, the use of *macrospargers* for oxygen supply, and medium replenishment. Implementing these parameters, among others, in a series of GMP production processes improved the titer yields by at least two orders of magnitude (2e9 PFU/mL) over previously reported values. The developed process was highly effective, repeatable, and robust, creating potent and genetically stable vaccine viruses and introducing new opportunities for application in other viral vaccine platforms.

## 1 Introduction

Recombinant vector vaccines produced in cell cultures have become a powerful tool to combat emerging infectious diseases. Among various viral vector platforms, recombinant vesicular stomatitis virus (rVSV) has emerged as a reliable backbone for vaccine development against outbreaking viral pathogens (Fathi et al., 2019). Notable successes have been achieved in the development of VSV-based vaccines against Ebola virus, HIV (Suder et al., 2018), measles, Lassa fever, and MERS (Henao-Restrepo et al., 2017; Kiesslich et al., 2020; Munis et al., 2020), and extensive studies have demonstrated the safety and efficacy of these vaccines in humans (Poetsch et al., 2019).

Previously, our institute designed an rVSV-based vaccine against the coronavirus SARS-CoV-2, commercially known as BriLife^®^ (Yahalom-Ronen et al., 2020). This innovative vaccine involved a recombinant replicating virus in which the surface VSV-G glycoprotein was replaced with the spike protein of SARS-CoV-2. Preclinical studies using the rVSV-ΔG-spike vaccine showed promising results (Yahalom-Ronen et al., 2020) and indicated the need to develop a robust and efficient cell-based GMP manufacturing process for further human clinical trials.

In recent decades, viral vaccines have been produced in continuous cell lines (Hilleman, 1990), such as adherently growing Vero cells (Milián and Kamen, 2015). The longstanding use of Vero cells and their regulatory approval by the World Health Organization (WHO) authorities for vaccine production made them the ultimate platform for manufacturing. Vero cells were used to produce several live and vector vaccines, such as Vaccinia, Polio, West Nile, and others (Kiesslich and Kamen, 2020). To increase virus production, bioprocesses have been adapted to use large-scale bioreactor systems, offering advantages such as sterility, automation, and robustness (Gallo-Ramírez et al., 2015). Previous studies have reported the development of bioprocesses for VSV-based vaccines using Vero cells adhered to microcarriers, such as Cytodex 1 (Kiesslich et al., 2020), or in suspension (Kiesslich et al., 2021). More specifically, the production of the rVSV-ΔG-spike vaccine in Vero cells using Cytodex 1 has recently been reported (Ton et al., 2023). However, these reports have indicated that further process improvement is needed to enhance titer yields. In this study, we are the first to describe the development of a bioprocess for VSV-based vaccine production in Vero cells using Fibra-Cel^®^ packed fixed-bed bioreactors. Fibra-Cel^®^ macrocarriers are composed of polyester fibers that provide a large growth area of 5 cm^2^ and three-dimensional protection against damaging shear forces, enabling the culture of millions of cells and maximizing viral yields. The bioprocess development of the rVSV-ΔG-spike vaccine was performed using WHO-approved Vero cells in serum-free conditions in packed-bed BioBLU^®^ 5p bioreactors (Eppendorf). Optimized parameters identified during development were integrated into the production process, resulting in enhanced virus titer yields of up to 2e9 plaque forming units (PFU)/mL, at least two orders of magnitude higher than previously reported. The safety and efficacy of the resulting substances were further assessed in human phase I/II clinical trials in a different study.

## 2 Materials and methods

### 2.1 Cell lines and culture media

Vero 10/87 cells (Barrett, 2009) were obtained from the World Health Organization (WHO) and grown in grade-A clean rooms. This cell line was initially cultured in serum-containing medium and gradually adapted to NutriVero FLEX-10 or FLEX-20 serum-free medium (specially formulated by Biological Industries Israel-Sartorius Ltd., product codes 05-068-1A and 05-069-1A, respectively). The use of serum-free medium is essential for vaccine production, as it eliminates the potential risks associated with serum-derived components. A GMP master cell bank and working cell banks were prepared from the adapted cells and used for process development and production. The cells passed all tests required by GMP guidelines for production in cell lines.

Vero E6 cells were purchased from ATCC and used for plaque-forming assays. Vero E6 cells were cultivated in DMEM (product code 01-055-1A, Sartorius) supplemented with 10% FCS (product code 04-121-1A, Sartorius), 2 mM L-glutamine (product code 03-022-1B, Sartorius), 0.1 mM NEAA (01-340-1B, Sartorius), and 0.5% (v/v) penicillin/streptomycin (product code 03-031-1C, Sartorius) at 37°C under 5% CO_2_.

### 2.2 Vessel preparation before cell inoculation

For experiments in Shake flasks and Celligen 310 glass bioreactors, Fibra-Cels (New Brunswick Scientific; M1292-9984) were weighed (1 gr, 230 pieces in shake flasks, and 25 gr, 5750 pieces in Celligen 310 glass bioreactor), mixed in PBS and autoclaved. Then, the PBS was replaced with fresh FLEX-20 (5 L), and the Fibra-Cels were transferred to the culture vessel. The vessels were equilibrated before cell inoculation by overnight incubation at 37°C, 5% CO_2_, and 60 rpm round agitation. Celligen 310 bioreactors were set to 37°C (using PT-100 sensor), 80 rpm, pH 7.2, and 50% dissolved oxygen (DO) using a Mettler Toledo electrode. Perfusion and titration systems were installed. After cell adsorption, the reactor was monitored for 5–7 days, and Fibra-Cels were sampled every day using a vacuum pump for cell count and biochemistry analyses.

For experiments conducted using the ready-to-use single-use GMP packed-bed BioBLU^®^320 bioreactor vessel (Eppendorf) containing 35,000 Fibra-Cels, each vessel was installed according to the manufacturer’s instructions and then equilibrated overnight with fresh FLEX-20 (3.5 L). Process conditions were set to 37°C, 90 rpm, pH 7.1, and dissolved oxygen (DO) of 50%. The pH set point was maintained by titration using a 7.5% bicarbonate solution (Sartorius).

### 2.3 Cell adsorption and growth on Fibra-Cels in BioBLU^®^320 bioreactors

For experiments in shake flasks, the cells were adsorbed to the Fibra-Cels for 90 min under 45 rpm agitation to allow at least 90% adsorption (data not shown). Then, the flasks were incubated for 6 days at 60 rpm agitation, and each day, approximately 50% of the medium was replaced with fresh medium. Each day, samples were collected to measure the number of cells on Fibra-Cels using the Alamar Blue assay as described earlier (Rosen et al., 2021).

For experiments in BioBLU^®^320 bioreactors, Vero cells were harvested from Multi-trays or the Xpansion^®^ system by washing with PBS and incubation with recombinant trypsin-EDTA solution (03-102-1B, Sartorius). The detached cells were stained using trypan blue solution (02-020-1A, Sartorius), counted in a Countess^TM^ cell counter (Invitrogen), and transferred to culture vessels containing Fibra-Cels (100,000 cells/Fibra-Cel at 80 rpm or as indicated in the text). The time needed to achieve >90% absorption of cells to Fibra-Cels varied among vessels: 60–90 min in shake flasks, 60 min in Celligen 310, and 45 min in BioBLU^®^320 bioreactors. After cell absorption, 70% of the culture medium was replaced with fresh FLEX-20 medium, and the cells were allowed to grow on the Fibra-Cels for an additional 6 days. 24 hours after inoculation, the perfusion system was initiated at an accelerating rate of 1.5–5 L/day. Medium exchange and additions of FLEX-10 supplements were performed as indicated in the text.

### 2.4 rVSV-ΔG-spike virus production in BioBLU^®^320 bioreactors

A stock of 200 vials containing aliquots of rVSV-ΔG-spike virus that passed the tests required by GMP guidelines for a viral production bank was used for production. All supplies used for GMP production passed the required tests, including purity, contaminants (Charles River Labs), genetic stability (IIBR), and sterility (HyLabs). The stock was stored at −80°C. Prior to infection, 70% of the culture medium was replaced with fresh FLEX-20 medium. Then, an aliquot of the virus stock was thawed (10 ± 5 min 20°C ± 4) and added to the bioreactors at a multiplicity of infection (MOI) of 0.1. During infection, the following sequence of agitation speeds was applied: 40 rpm for 5 min, 20 rpm for 20 min (or as indicated in the text), and then a gradual cascade elevation back to 70 rpm. Six hours after infection (at the time of viral entry into the cells), 70% of the culture medium was replaced. The production process was monitored for a total of 48 h, with samples taken periodically for in-process controls (IPCs). As part of the process development, some experiments included two 10% supplements (350 mL each) of FLEX-10 at 8 and 24 h post-infection. After 48 h of production, the supernatant culture medium was harvested into 5 L Flexboy bags (Sartorius) and transferred for downstream clarification.

### 2.5 In-process and postproduction analyses

Supernatant samples were taken for in-process control (IPC) analyses at different time points during the process: 5 min, 2 h, 6 h, 8 h, 24 h, 36 h, and 48 h. The samples were examined in Cobas c111 (Roche) for specific metabolites and waste products, such as glucose, lactate, glutamate, glutamine, ammonia, and lactate dehydrogenase (LDH) and PFU assay, as described in the next paragraph.

At the harvest time point (48 h), the vessel integrity was broken, and the number of cells attached to the Fibra-Cel was assessed using Alamar Blue as described earlier (Rosen et al., 2021). Fibra-Cels with adsorbed Vero cells were stained with calcein AM solution (C1359, Merck) and visualized by Nikon TS2 fluorescence microscopy, and the images were analyzed in ImageJ.

Viral titer yields were determined using a PFU assay. Briefly, Vero E6 cells were cultured (0.75-1e6 cells/well) in 6-well plates and incubated overnight at 37°C. Then, the plates were washed with PBS, and the diluted samples were implanted (0.2 mL) in culture wells. After 60 min of incubation with gentle agitation, an overlay medium containing 0.8% Tragacanth (G1128, Merck) in MEM containing 2% FCS, 2 mM glutamine, and 1% NEAA was added to each well. After 3 days of incubation (37°C, 5% CO_2_), the cells were washed with PBS and fixed with 0.1% crystal violet solution (Sartorius), and the number of plaques was counted. The virus titer was calculated by multiplying the number of plaques by the dilution factor and by 5 (sowing volume factor) to obtain the number of PFU per mL.

Host-cell protein analysis was conducted to measure the amount of host proteins derived from Vero cells. Samples from the harvested substances were tested for residual proteins using a Host-Cell Protein ELISA Kit (Cygnus Technologies, #F500) following the manufacturer’s instructions.

### 2.6 Genetic stability analysis of the viral vaccine post-production

Whole-genome sequencing analysis was performed to monitor modifications in the viral genome post-production. Briefly, the SMARTer Pico RNA Kit (Clontech) was used for library preparation, and then the Illumina MiSeq platform, with a paired-end mode and a read length of 150 × 2 nucleotides, was employed for whole-genome sequencing, producing 4,005,602 reads per sample on average. FastQC, TrimGalore, and Cutadapt were applied for quality control analysis and read trimming. Read mapping against the reference rVSV-SARS-CoV-2 was performed using Bowtie 2, yielding an average of ∼2,000,000 reads across all the samples. These steps resulted in an average coverage of ∼6,000x across all the samples. Variant calling was performed using SAMtools with the default parameters. High-resolution counting of the occurrence of each base at each position along rVSV-SARS-CoV-2 was performed by igvtools with the parameters ‘count’, -w 1 and -bases.

We utilized the ggfortify package in R to construct a Principal Component Analysis (PCA) graph. The input data included the counting of the occurrence of each base at each position of interest along the genome. The ggfortify package enhanced ggplot2, providing a clear visual representation of data patterns and clusters contribution to each principal component.

### 2.7 Statistics

The Prism 5.01 (GraphPad) software was used for statistical analysis, using nonparametric Wilcoxson tests. Differences were considered significant at *p* < 0.05.

## 3 Results

### 3.1 Cell adsorption and growth on Fibra-Cels

**FIGURE 1:**
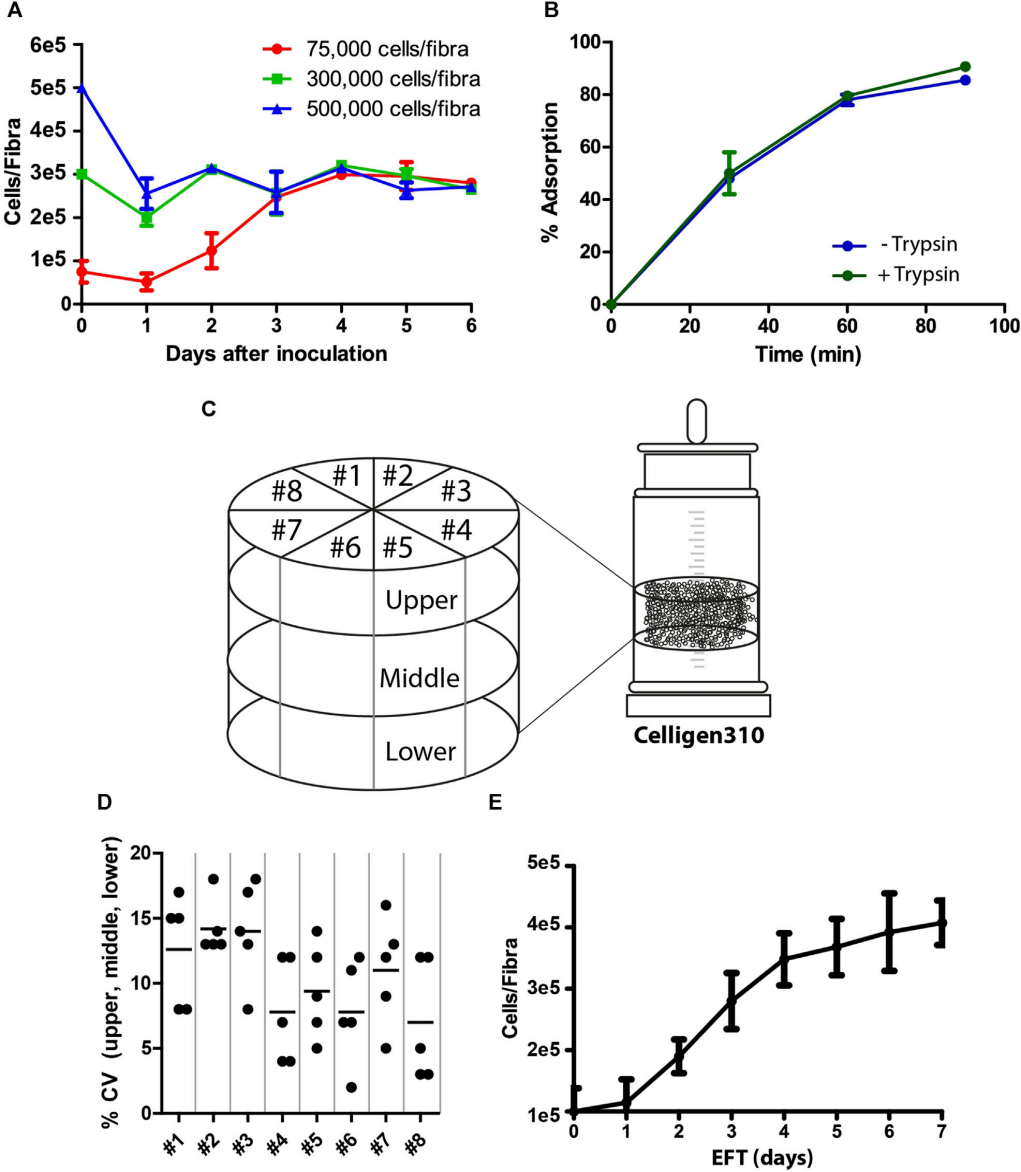
Cell adsorption and growth on Fibra-Cels. **(A)** Growth curves of cells on Fibra-Cels in shake flasks for 6 days after inoculation with 75,000 (red), 300,000 (green), and 500,000 (blue) cells per Fibra. **(B)** Adsorption percentages over time of cells on Fibra-Cels in shake flasks after trypsin EDTA treatment, without washing (Trypsin +) or after washing with PBS (Trypsin -). **(C)** An illustration of the experimental design in the Celligen 310 glass bioreactor. The vessel was virtually divided into three layers (upper, middle, and lower) and eight pieces (#1- #8). Five Fibra-Cels were sampled from each area, and the number of adsorbed cells was determined. **(D)** A dot plot showing the CV percentiles of five Fibra-Cel samples in each layer: upper, middle, and lower from eight pieces. **(E)** A cell growth curve on Fibra-Cels in Celligen 310 vessels over 7 days. *n* = 5.

The process of viral vaccine production involved two major steps: cell adsorption and propagation, followed by vaccine production. The goal of the first step was to prepare highly functional competent Vero cells that would facilitate optimal virus production with high yields. Because Fibra-Cels have a large cell capacity, we examined the optimal ratio of cells to Fibra-Cels at the time of adsorption to provide the maximal effective cell biomass. To that end, we used a shake flask as a small-scale model and examined the adsorption and growth of elevated cell amounts (75,000-500,000 cells/Fibra-Cel) over 6 days. We detected that the inoculation of 75,000 cells/Fibra-Cel resulted in a gradual increase in cell numbers to 300,000 cells/Fibra-Cel until day four, and that value remained the same until day six. The inoculation of 300,000 and 500,000 cells/Fibra resulted in the same cell capacities after 6 days (Figure 1A), meaning that there was a limit to cell growth in the Fibra-Cels under these specific conditions and that the optimal number of cells for inoculation was 75,000-100,000 cells.

During the preparation of the cell inoculum, surface-attached Vero cells were peeled off using recombinant trypsin and transferred for further adsorption on Fibra-Cels in the bioreactor vessel. Since trypsin is usually used for cell dissociation, it could impair the initial cell adsorption efficiency. To test this hypothesis, we examined cell adsorption on Fibra-Cels at a ratio of 100,000 cells/Fibra-Cel directly after treatment with trypsin or after washing it off. As shown in Figure 1B, the presence of trypsin in the inoculum did not significantly influence the efficiency of cell adsorption on Fibra-Cels within 90 min of incubation. Therefore, the cell centrifugation and washing steps before cell inoculation were eliminated in the remaining experiments to simplify the process. To remove trypsin after cell adsorption, 70% of the culture medium was replaced, and 24 h later, the perfusion system was turned on.

### 3.2 Cell scattering and adsorption uniformity inside the bioreactor

BioBLU^®^320 vessels are predefined hermetically sealed single-used bioreactors; therefore, it was not possible to monitor cell scattering and adsorption uniformity inside a bioreactor during the process. To overcome this limitation, a former version of the Cellgen310 glass bioreactor was used as a model, in which a vacuum pump was used to sample Fibra-Cels from various parts of the vessel during the experiment. Fibra-Cels were sampled at different time points from the upper, middle, and lower parts and from eight locations around the bioreactor vessel using a vacuum pump (see illustration in Figure 1C). Each designated area was sampled five times in each layer, so overall, there were five repeats from each piece and from each layer. The mean number of living adsorbed cells on Fibra-Cels at the end of the process was 3e5-5e5 cells with minor variations of less than 20% CV between tested areas (Figure 1D), indicating uniform and equal distribution of cells over the vessel. The adsorbed cells inside the vessel were grown for 7 days with 50% medium replenishment every day, reaching maximal levels after 6–7 days (Figure 1E). These results were consistent with the results found in shake flask experiments, suggesting that the optimal period of cell growth inside the bioreactor is 6 days.

### 3.3 Process optimization

**FIGURE 2:**
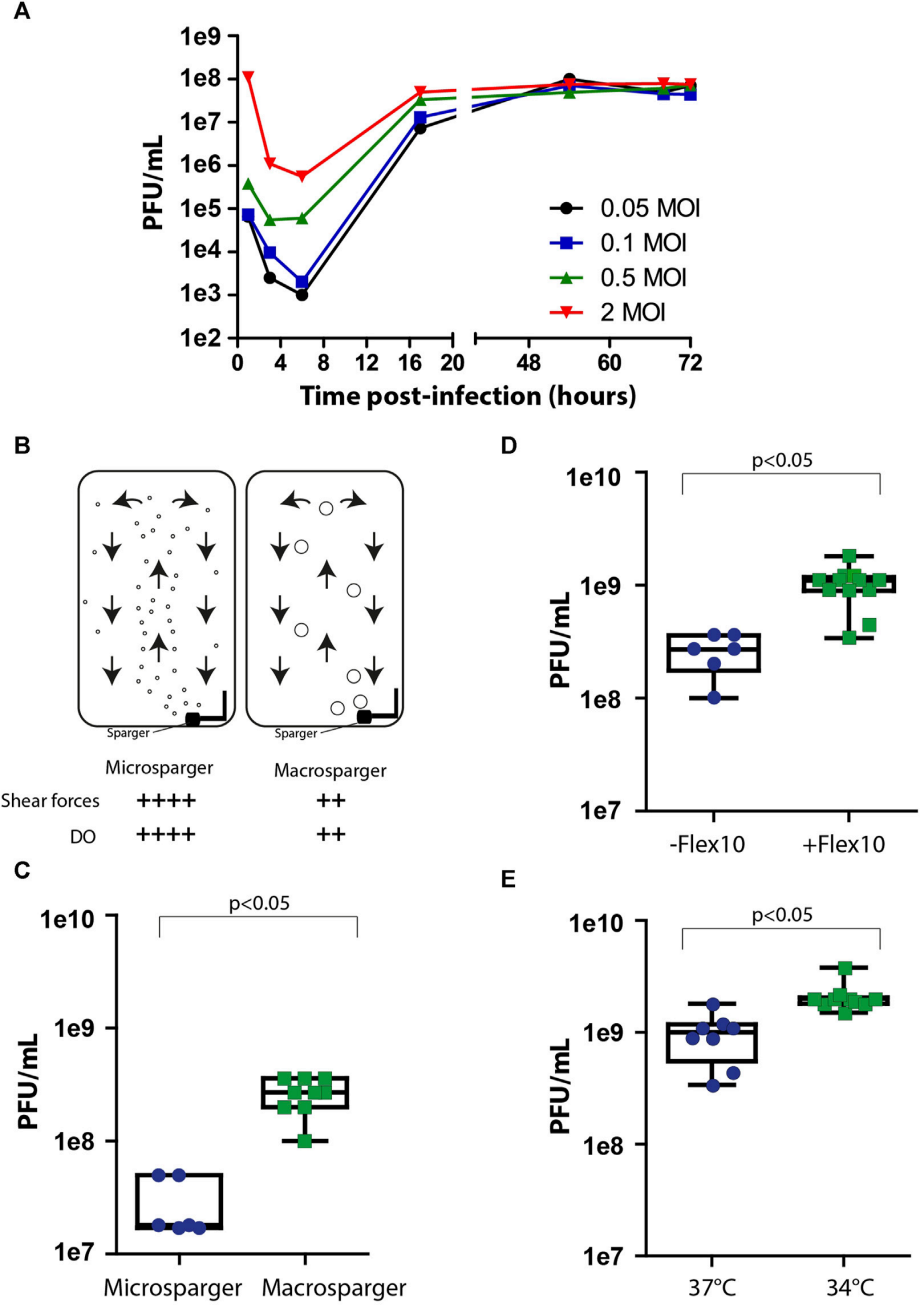
Process optimization. **(A)** Virus production kinetics over time in shake flasks after infection with MOIs of 0.05 (black), 0.1 (blue), 0.5 (green) and 2 (red). **(B)** An illustration of micro- and macrosparger bioreactors. Microsparger bioreactors are characterized by high oxygen dissolving efficiency and shear forces (++++), whereas macrosparger bioreactors are characterized by lower oxygen dissolving efficiency and lower shear forces (++) (upper panel). **(C–E)** Box plots of virus production yields in microsparger or macrosparger bioreactors **(C)**, after two additions of 350 mL FLEX-10 medium, 8 and 24 h post-infection **(D)**, and at 37°C or 34°C **(E)**. *n* = 8-10. *p* < 0.05 was tested by the nonparametric Wilcoxon test.

After achieving maximal cell biomass inside the bioreactor, the cells were infected with the viral vaccine. One of the key factors affecting virus production yields in the process is the multiplicity of infection (MOI), which is the number of infectious particles per cell. Since different types of viruses require different MOI ratios in order to optimally propagate in the process, it is essential to individually identify MOI ratios that maximize the production yields. Inappropriate MOI ratio could lead to massive cell death, instability in culture steady state, and the abrogation of subsequent generations of virus production. To examine the optimal range of MOI in the process, cells were grown on Fibra-Cels in shake flasks and, after 6 days, infected with MOIs of 0.05, 0.1, 0.5, and 2. Supernatant samples were collected at different time points during 72 h of incubation and assessed in the PFU assay. We determined that the number of infectious viruses decreased to minimal levels after 6 h–when most of the infectious viruses had entered the cells. Then, there was a logarithmic increase in virus titer for the next 10 h, reaching maximal levels at 48–50 h post-infection (Figure 2A). All MOI ratios resulted in the same virus production titers, indicating that a wide range of MOI values can be used in the process. However, considering the initial viral seeding inoculum, high MOI levels (0.5-2 MOI) have led to lower yield returns (Figure 2A). Thus, the optimal yield returns in the process were achieved using 0.05 and 0.1 MOI (Figure 2A). These results were consistent with our previously reported findings using the Ambr15^®^ system (Rosen et al., 2022).

After accumulating basic parameters of the process in shake flasks and glass bioreactors, such as the inoculation ratio, time of cell growth for optimal cell biomass, and MOI, we continued the process optimization using packed fixed-bed ready-to-use BioBLU^®^320 bioreactors. These vessels are commercially supplied with two types of gassing spargers to provide a constant level of dissolved oxygen (DO) in the process: *microspargers*, which produce microbubbles with high oxygen dissolving efficacy but also high shear forces, and *spargers*, which produce macrobubbles with relatively lower oxygen dissolving efficacy and lower shear forces (Figure 2B). To compare the effects of sparging on process yields, we assessed virus production using vessels with micro- or macrospargers. The virus production yields were ten times higher in the macrosparger vessel than in the microsparger (from 3e7 PFU/mL to 5e8 PFU/mL on average) (Figure 2C), suggesting that the high shear forces caused by using the microsparger impaired process productivity. The remaining experiments were performed in macrosparger vessels.

During cell growth, the perfusion system was turned on. However, we turned it off during the virus production phase to prevent the dilution of virus titers. Unfortunately, the absence of medium refreshments during production can hamper cell productivity because of limited medium nutrients. To overcome this, we exploited the extra volume in the vessel to supply two additions of Flex-10 medium (350 mL each) 8 and 24 h post-infection and examined the added value on virus production yields. We found that these two medium supplements increased the mean virus titers from 5e8 PFU/mL to 1e9 PFU/mL (Figure 2D).

Another parameter previously reported in the literature to improve the productivity of the virus production phase is temperature reduction to 34°C, which forces the cells to shift their resources toward the production of virus particles (Paillet et al., 2009; Elahi et al., 2019; Gélinas et al., 2019). To assess the effect of temperature shift on virus titer in our system, we compared virus production at two temperatures: 34°C and 37°C, in addition to Flex-10 medium supplements, as indicated above. As shown in Figure 2E, the reduction in temperature resulted in a twofold increase in infectious virus yields compared to that at 37°C.

To conclude, basic process parameters were optimized, including inoculation ratio, time of cell growth for optimal biomass, MOI, infection kinetics and virus production, resulting in an overall increase of 2 orders of magnitude in virus titer yields.

### 3.4 In-process and postproduction analyses of GMP bioprocess vaccine production

**FIGURE 3:**
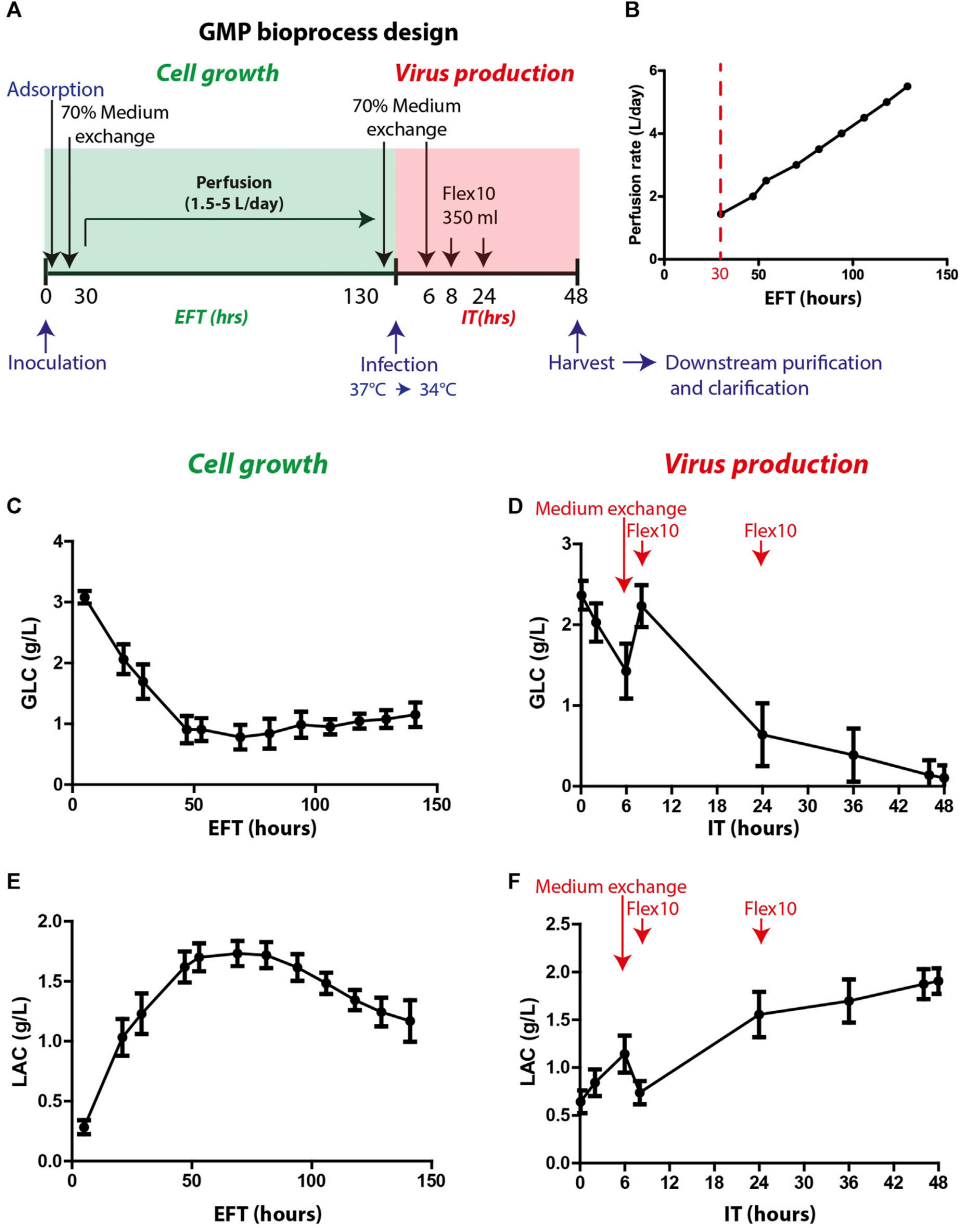
GMP bioprocess production and the kinetics of glucose and lactate levels in the process. **(A)** An illustration of the timeline of the developed bioprocess: cell growth (green) and virus production (red) phases. Cells were inoculated and absorbed on Fibra-Cels in bioreactors. After >90% adsorption, 70% of the culture medium was replaced, and the perfusion system was turned on. Cells were grown for 6 days under constant perfusion, and then 70% of the medium was exchanged before infection with the virus. Six hours post-infection, another 70% of the medium was replaced, and two FLEX-10 supplements (350 mL each) were added 8 and 24 h post-infection. After 48 h, the culture supernatant was harvested and transferred to downstream purifications and clarifications. **(B)** Perfusion rates over 6 days of cell growth. **(C–D)** Glucose levels during cell growth (estimated fermentation time, EFT) and virus production (infection time, IT). **(E–F)** Lactate levels during cell growth and virus production. *n* = 30. Red arrows show the time of medium exchange and additions during the virus production phase.

The optimized process parameters, as determined in the process development, were employed in thirty proceeding bioprocess production cycles. These production cycles were adapted to the GMP requirements and specifically to the Annex I chapter of the European Pharmacopeia for phase I and II clinical trials and were conducted under aseptic clean room conditions. The final bioprocess format included two phases: cell growth and virus production (as illustrated in Figure 3A). The cell growth stage included cell expansion in the Xpansion^®^ system and multi-trays and inoculation and adsorption on FibraCels inside the BioBLU^®^320 bioreactors. The cells were grown for 6 days in Flex-20 medium with constant medium replacement using the perfusion system at an increased rate from 1.5 L/day to 5 L/day (Figure 3B). In the virus production phase, the temperature was shifted to 34°C, and there were two 70% medium exchanges: before virus infection and 6 h post-infection, as well as two Flex-10 supplements at 8 and 24 h post-infection. After 48 h of virus propagation, the culture supernatant was harvested and transferred to downstream purification.

To assess cell functionality and productivity, various parameters were monitored during and at the end of the cell growth and virus production phases. These parameters included glucose consumption, lactate secretion and consumption, lactate dehydrogenase (LDH) secretion (indicating cell death), and host cell protein (HCP) levels (indicating host impurities).

Glucose and lactate were used as indicator parameters to monitor process progression during the cell growth phase (Figures 3C, E). The glucose consumption rate increased, leading to a substantial reduction in glucose levels during the first 50 h of cell growth (estimated fermentation time, EFT) (Figure 3C). The increase in perfusion rate stabilized the glucose levels (0.5–1.5 g/L) for the remaining growth phase (Figure 3C). Consequently, the lactate levels increased during the first 50 h of the process and then decreased after 100 h (Figure 3E) when the cells began to consume lactate as a secondary carbohydrate source. Prior to infection, perfusion was stopped, and 70% medium exchange was performed to elevate glucose levels to 2–2.5 g/L. After infection, the vessel temperature was reduced to 34°C, and the agitation speed was decreased to 40 rpm for 5 min to allow equal distribution of the virus throughout the vessel. Next, the agitation speed was further reduced to 20 rpm for an additional 20 min to enable virus adsorption and penetration to the cells and then gradually increased to 70 rpm. During the first 6 h after infection time (IT), a sharp reduction in glucose levels was observed (Figure 3D). Thus, to increase glucose levels, an additional 70% medium exchange was performed after most of the viral particles entered the cells. To further support the culture during production, Flex-10 medium was added twice, at 8 and 24 h post-infection, which contributed to stabilizing the glucose levels for the remaining virus production phase (Figure 3D). The pattern of lactate levels in the process was opposite to that of glucose (Figure 3F). To summarize, in-process analysis of glucose and lactate levels demonstrated high uniformity and repeatability, which are essential to maintain high quality and consistency of the vaccine production process.

**FIGURE 4:**
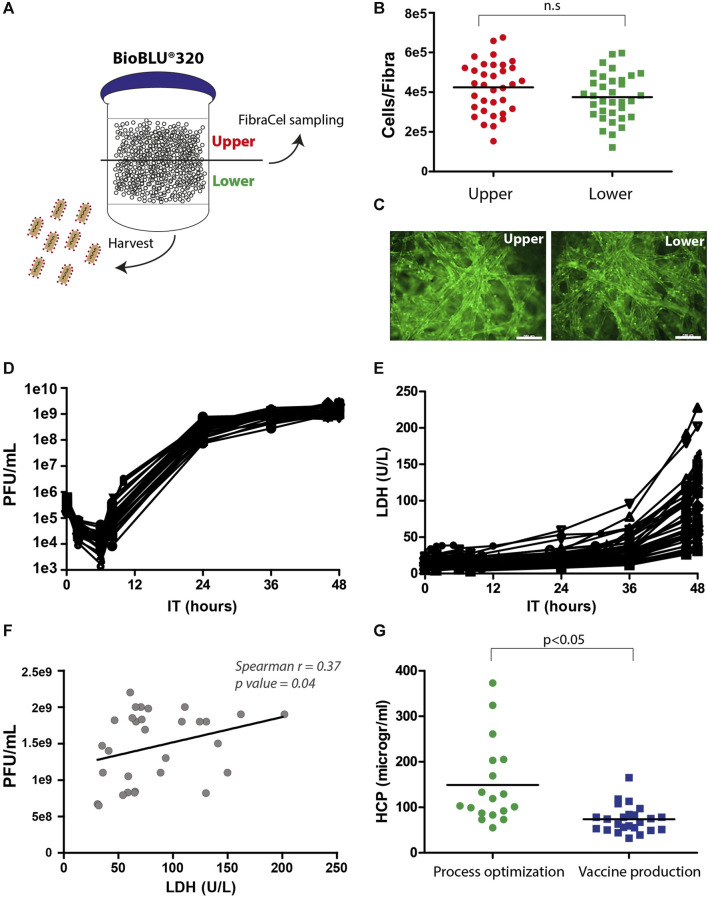
Postprocessing analyses of living cells, LDH, HCP and virus titers. **(A)** An illustration of virus harvest and Fibra-Cel sampling from the upper and lower sides of the BioBLU^®^320 bioreactor vessel. **(B)** A dot plot showing cells per Fibra-Cel on the upper (red) and lower (green) sides of the bioreactor vessel. *n* = 30, not significant (n.s.) according to the Wilcoxon nonparametric test. **(C)** A fluorescence microscopy image showing cells stained with calcein on Fibra-Cels sampled from the upper and lower sides of the bioreactor vessel. Bar scale 200 µm. **(D)** Virus titer kinetics over 48 h of production. **(E)** LDH levels over 48 h of production. **(F)** A correlation dot plot with a linear curve showing a significant correlation between virus titers and LDH levels at the end of the process (Spearman *r* = 0.37, *p*-value 0.04). *n* = 30. **(G)** HCP levels in harvest supernatants from bioreactor vessels during the process optimization and development phase and the vaccine production phase. *n* = 18–30.

Samples from the upper and lower vessel sides were collected and measured for viability in the Alamar blue assay and fluorescence microscopy, and the supernatant was examined for virus yields in PFU assays (Figure 4A). The number of live cells on Fibra-Cels ranged between 2e5 and 7e5 (4e5 cells on average), and the production yields ranged between 1e9 and 3e9 PFU/mL (not shown). There was no significant difference between the upper and lower sides of the vessel (Figures 4B, C). Although we identified a correlation between living cells and production yields, it reached saturation at >350,000 cells/Fibra-Cel and became nonsignificant thereafter (data not shown). This suggests that a high number of living cells at the end of the process would not necessarily predict high titer yields. These results were consistent with our previously reported findings using the Ambr15^®^ system (Rosen et al., 2022).

LDH is commonly accepted as a marker of cell death, as its release to the medium indicates cell degradation (Kumar et al., 2018). To study the effect of virus propagation on cell lysis, during the process, supernatant samples were collected at different time points and tested for virus titer and LDH levels. Figure 4D describes the kinetics of virus infection and propagation during the process; 6 h post-infection, there was a reduction in the number of infectious particles as most of the viruses entered the cells, followed by logarithmic production and an increase of 3.5 logs in virus titers within 24 h and an additional 0.5 log increase between 24 and 48 h post-infection. The mean yields of all the processes were 2e9 PFU/mL. In addition, we identified a substantial elevation in LDH levels after 36 h and observed that the final LDH levels varied between processes (Figure 4E). This variation in LDH levels at the end of the processes was linearly correlated with the virus titer (Spearman *r* = 0.37, *p*-value = 0.04) (Figure 4F). This suggests that the differences in LDH levels at the end of the process reflect differences in virus titers, indicating that LDH can serve as a marker of cell lysis caused by virus propagation.

HCP are process-related protein impurities derived from the host cells. Although HCPs can be removed during downstream purification, there is an advantage to end the production phase with as low HCP levels as possible. An effective way to reduce the HCP levels is by medium replacement during the process. Indeed, we identified, during process optimization, that processes without medium exchanges had higher HCP levels, whereas processes with medium replacement, 8 hours post-infection, had lower HCP levels (50–400 mg/mL) (Figure 4G, “process optimization”). As a result, the preceding production processes included medium exchange that was preformed after 6 hours and additions of two medium supplements after 8 and 24 h (Figure 3A), leading to reduced HCP mean levels to ∼80 mg/mL (Figure 4G, “vaccine production”).

**FIGURE 5:**
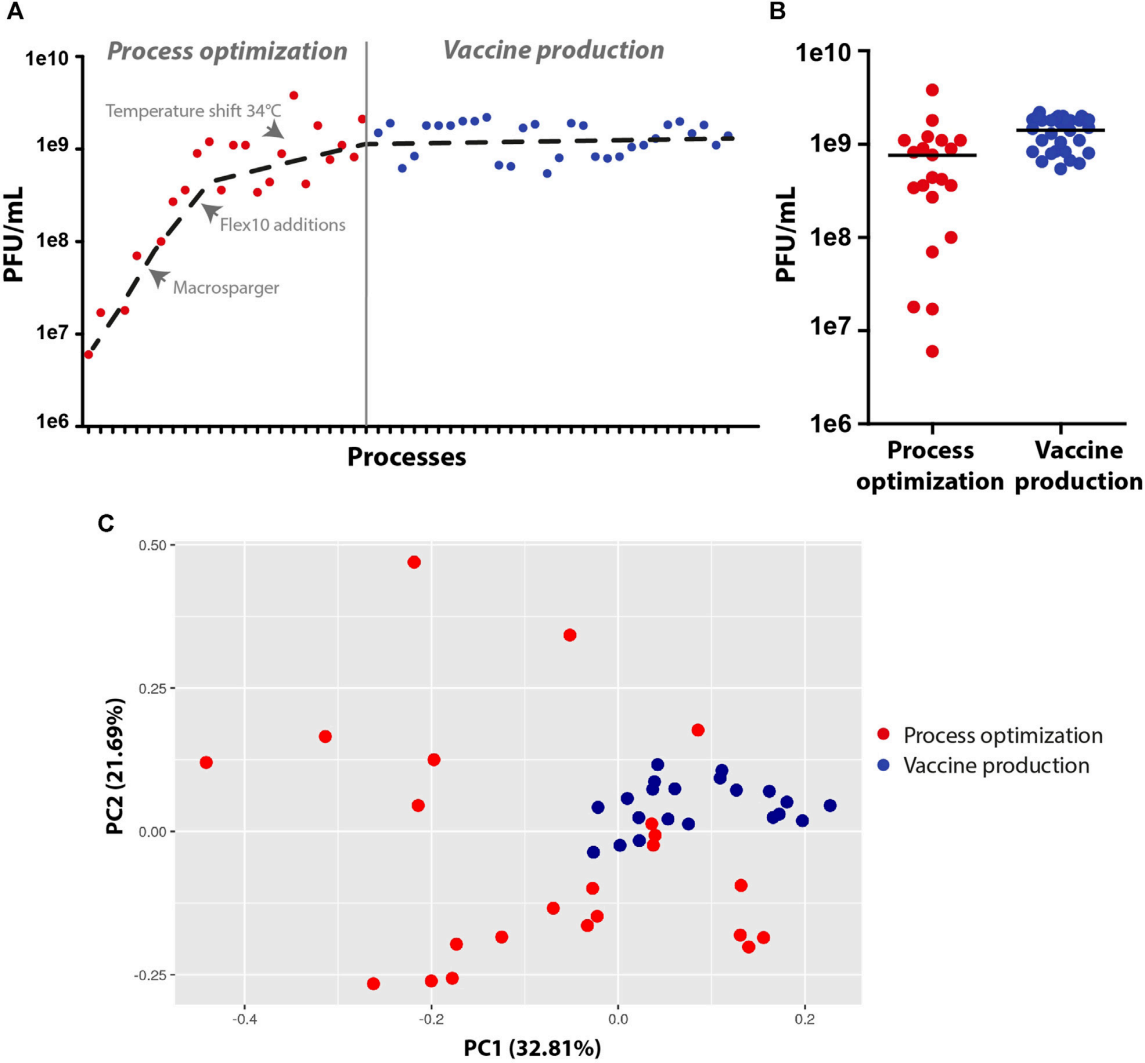
Virus production yields and genetic stability at the process optimization and vaccine production stages. **(A,B)** Dot plots of virus titer yields at the process optimization and vaccine production stages. **(C)** Principal component analysis (PCA) of the virus vaccine genome, demonstrating variations in genome sequences at the development and optimization stages (scattered red dots) and high similarity in genome sequences at the vaccine production stage (clustered blue dots) (*n* = 20-23).

To summarize, during the process development and optimization, the virus titer yields increased by at least two orders of magnitude (1e7 to 2e9 PFU/mL), and the vaccine production runs were robust and reproducible, leading to an average of approximately 2e9 PFU/mL with a small standard deviation (Figures 5A, B). To ensure that all of the vaccine production batches were similar and robust, samples from the harvest were sequenced to detect modification in the genome, and the data were analyzed using principal component analysis (PCA). PCA simplifies the complexity of the varying nucleotide frequencies along the genome, reveals important patterns and might correlate with changes in the process conditions. The points representing samples from final vaccine production were tightly clustered, indicating high similarity and robust genetic stability. In contrast, points representing samples prior to final optimization at the development stage were more dispersed, indicating less cohesion and more variable genetic patterns (Figure 5C). Only fourteen genetically stable mutations occurred, which accounted for only 0.09% of the genome. Of these mutations, nine became dominant, while two mutations, W64R and H66R, showed alternative occurrences. Specifically, nine mutations were detected in the S open reading frame, which encodes the spike protein, and five mutations were identified in the N and L genes of the backbone. Overall, the rVSV-SARS-CoV-2 vaccine acquired a minimal number of mutations, demonstrating that the conditions of the process were optimal for cell growth and virus propagation in a robust and reproducible production batch.

## 4 Discussion

**FIGURE 6:**
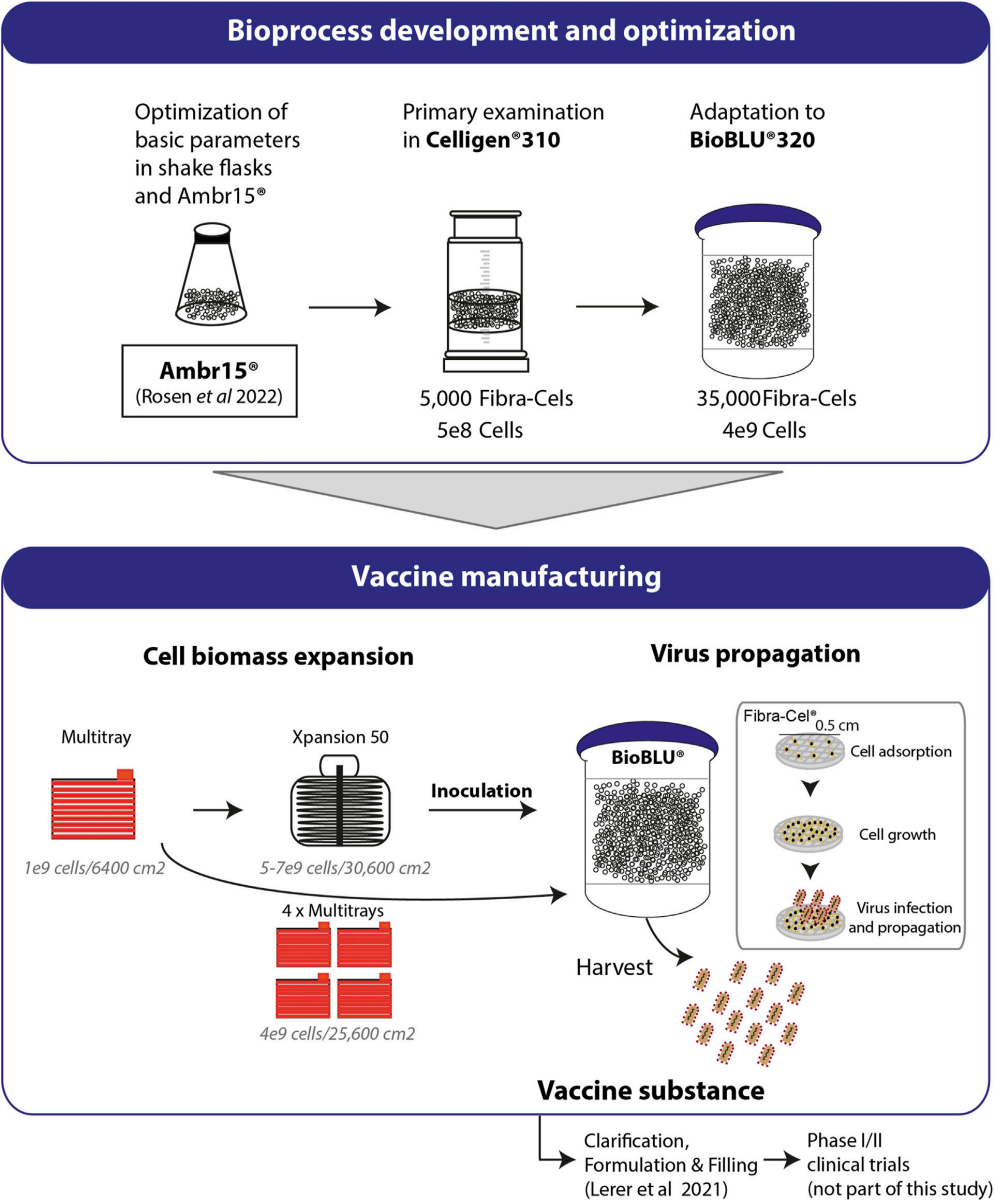
Process development and production. Basic parameters of the process were optimized using shake flasks and Ambr15^®^ (Rosen et al., 2022). Cell adsorption and growth were examined in Celligen^®^ 310 using 5000 Fibra-Cels inoculated with 5e8 cells. Further adaptations and optimizations were performed in a packed fixed-bed BioBLU^®^320 bioreactor containing 35,000 ready-to-use Fibra-Cels. The parameters examined included cell adsorption and growth, virus infection and harvest yields. Optimized parameters were integrated in a final GMP bioprocesses format including Vero cell expansion in four Multi-trays (4e9 cells/25,600 cm^2^) or in the Xpansion50^®^ system (5-7e9 cells/30,600 cm^2^) and inoculation in BioBLU^®^320 vessels. The cells were infected with the virus and produced the vaccine. The vaccine substance was further clarified downstream, formulated, and administered by injection to participants in a phase I/II clinical trial (not part of this study).

In this study, we described the development and optimization of rVSV-ΔG-spike vaccine production in packed-bed bioreactors. Figure 6 summarizes the optimization progress, started with shake flasks as model vessels and validations performed in mini-vessels of the Ambr15^®^ system (recently reported, Rosen et al., 2022), and continued with the Celligen 310 glass bioreactor and single-use packed-bed BioBLU^®^ 5p bioreactors (Figure 6). The developed bioprocess included two major steps: cell biomass expansion and virus propagation. The cells were expanded in Multi-trays and Xpansion^®^ systems to achieve a large inoculum and transferred to single-use BioBLU^®^ 5p bioreactors, where vaccine production took place (Figure 6). The vaccine substances from the process were transferred to downstream purification, clarification and formulation (Lerer et al., 2021) and further investigated in phase I and II clinical trial participants (in a different study) (Figure 6).

Different parameters were investigated for their effect on cell function and productivity for virus production. Cell viability and scattering throughout the vessel cabin and total cell biomass are significant factors determining the efficacy and productivity of the process. One effective way to increase functional virus production is to increase the number of adsorbed cells on Fibra-Cels. However, at a certain point, overloading had a redundant effect. We determined that the optimal adsorption ratio for the process needs to be between 75,000 and 100,000 cells per Fibra-Cel. At that density, the cells were highly distributed all over the bioreactor vessel and uniformly adsorbed. Importantly, we found that macrosparger bioreactors that produced macrobubbles with reduced shear forces were beneficial to cell viability and function in the process but increased titer yields by one order of magnitude.

Another important parameter that dictates the efficacy of virus production is MOI. Infection with high MOIs would lead to more infected cells and therefore to more intense and efficient processes. However, too many infectious particles would have a direct influence on cell viability and growth cycles throughout the process. It has been reported that an excessively high MOI produces defective-interfering particles, resulting in reduced virus titers (Linder et al., 2021). Thus, 0.1 MOI has been conventionally used in these studies (Elahi et al., 2019; Gélinas et al., 2019). However, the exact number of cells at the time of infection cannot be measured in our process settings using a packed-bed bioreactor, and therefore, the precise MOI cannot be calculated. Therefore, it was essential to identify the working range in which the process would remain effective. We determined that at a wide range of MOIs of 0.05–0.5, the production efficacy was similar, indicating that there is a degree of flexibility in the process at that specific range.

Significant improvement in production yields came from the adjustment and timing of the perfusion rate and medium exchange regime in accordance with the glucose levels throughout the process. We monitored glucose levels in the cell growth phase and adjusted the perfusion at an elevated rate from 1.5 to 5 L/day to retain glucose values above critical levels of 0.5 g/L. In addition to glucose consumption, we observed a reduction in lactate levels because of the lactate metabolic switch from secretion to consumption as a secondary carbohydrate source after 3 days of growth (Figure 4D). This lactate metabolic switch has previously been described (Hartley et al., 2018), suggesting that lactate is actively transported into cells at low extracellular pH. However, under a controlled pH environment, lactate uptake was much more gradual and moderate (from 2 g/L to 1 g/L); therefore, cell nutrition was maintained, providing high viability and functionality. Before infection, perfusion ceased, and 70% of the culture medium was replaced to resupply nutrients to the culture, as reflected in the renewed elevation of glucose levels. However, this alone was not sufficient to support the culture for 48 h of production. To better support the production period, we exploited the time window in which most of the virus entered the cells and added another 70% medium replaced 6 h post-infection. This allowed maximal culture refreshment with a minimal loss of virus titer. Medium replenishment also contributed to the reduction in HCP levels–process-related protein impurities produced by the host cells, as required by the European pharmacopeia of GMP production. To increase virus titer yields even more efficiently, we exploited the vessel net volume more efficiently and added two Flex-10 supplements (350 mL each) at 8 and 24 h post-infection.

Another significant improvement in the process was achieved in temperature reduction to 34°C at the time of production. This forced the cells to shift their resources toward the production of virus particles, as we previously showed using the Ambr15 system (Rosen et al., 2022) and as others have reported (Paillet et al., 2009; Gélinas et al., 2019). Moreover, using the Ambr15 system, we showed that the reduction in production temperature to 34°C prolonged cell viability and reduced LDH and HCP release (Rosen et al., 2022).

Throughout virus production and at harvest, LDH levels were measured as an indicator of cell viability. We determined that LDH concentrations remained low in the process and elevated only close to the end of production at the time that most of the viruses were released from the cells. LDH levels were correlated with virus yields, suggesting that higher LDH levels are accompanied by increased virus titers and can mark an increase in total production yields.

To conclude, in this study, we presented a comprehensive view of bottom-to-top development and optimization of the bioprocess to produce the rVSV-ΔG-spike vaccine in Vero cells using Fibra-Cel macrocarriers. Each parameter in the process was examined separately and in combination, leading to a significant increase in virus titer yields of 2e9 PFU/mL, 2 orders of magnitude higher than previous reports using microcarriers. The developed process was adapted to the GMP requirements of the European Pharmacopeia. The developed process was highly genetically stable with minor variations in the genomic sequence, indicating that the process was highly effective, robust, and repeatable, introducing new opportunities for use in other viral vector vaccine platforms.

## Data Availability

The original contributions presented in the study are publicly available. This data can be found here: SRA database accession number—PRJNA1056526.
